# Supplementary material for: High-Altitude Hypoxia Exposure Induces Iron Overload and Ferroptosis in Adipose Tissue
Source: Antioxidants (Basel). 2022 Nov 29;11(12):2367. doi: 10.3390/antiox11122367 (PMC9774922; doi:10.3390/antiox11122367)
Supplement: Supplementary file 1 [file antioxidants-11-02367-s001.zip › antioxidants-1995150-supplementary.pdf]

Supplementary data

Table S1. Primers used for quantitative real-time PCR

Gene	Forward sequence (5'-3')	Reverse sequence (3'-5')
CCL2	ACATTCGGCGGTTGCTCTAG	ACATCCTGTATCCACACGGCAG
CD11c	CTGGATAGCCTTTCTTCTGCTG	GCACACTGTGTCCGAACTC
CHAC1	CTTGGTGGCTATGACACTAAGG	CCTCGGCAAGCAAGGATCTG
FTH	GATCAACCTGGAGTTGTATGCC	GATATTCTGCCATGCCAGCTTC
FTL	CAGTCTGCACCGTCTCTTCG	GTCATGGCTGATCCGGAGTAG
GPX4	CATGCACGAATTCTCAGCCA	CATATCGGGCATGCAGATCG
HMOX1	TGACACCTGAGGTCAAGCAC	TCTCTGCAGGGGCAGTATCT
IL1β	AGCCCATCCTCTGTCACTCA	TGTCGTTGCTTGGTTCTCCT
IL6	CTCTGCAAGAGACTTCCATCCA	CGTGGTTGTCACCAGCATCA
iNOS	GGAGTGACGGCAAACATGACT	TCGATGCACAACTGGGTGAAC
SLC7A11	ATCGGGACTGCTAATGAGAATT	CTATTTTACCACCATCAGTGCG
TFR	TGATTGTTAGAGCAGGGGAAA	ATGACTGAGATGGCGGAAAC
TNFα	TCCCAGGTTCTCTTCAAGGGA	GGTGAGGAGCACGTAGTCGG
18S rRNA	GCCCTCCAATGGATCCTCGTTA	GAAACGGCTACCACATCCAAGG

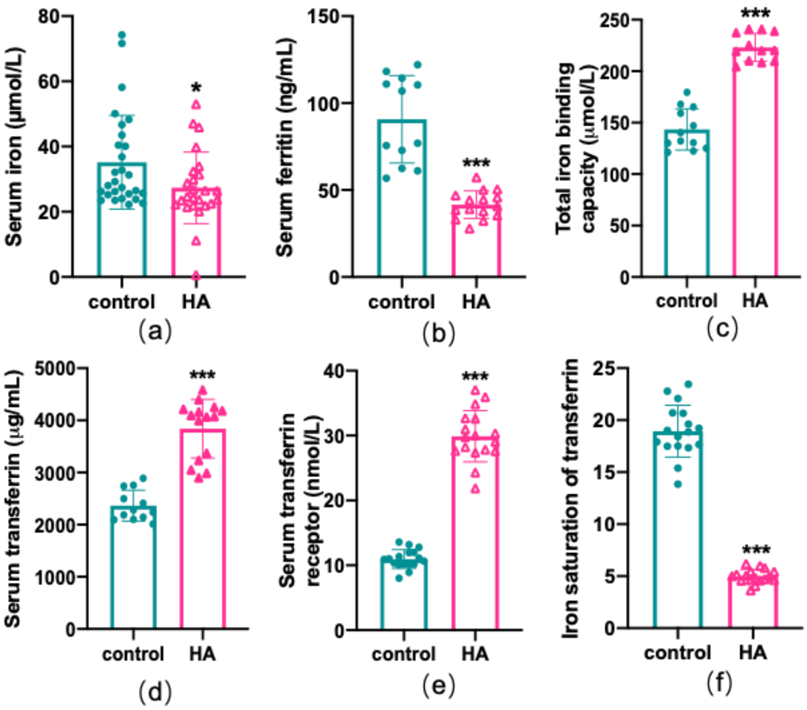

Figure S1. Serum iron parameters are altered during high-altitude hypoxia exposure.
Data are presented as the mean ± SEM. * $p < 0.05$, ** $p < 0.01$, and *** $p < 0.001$, versus control.

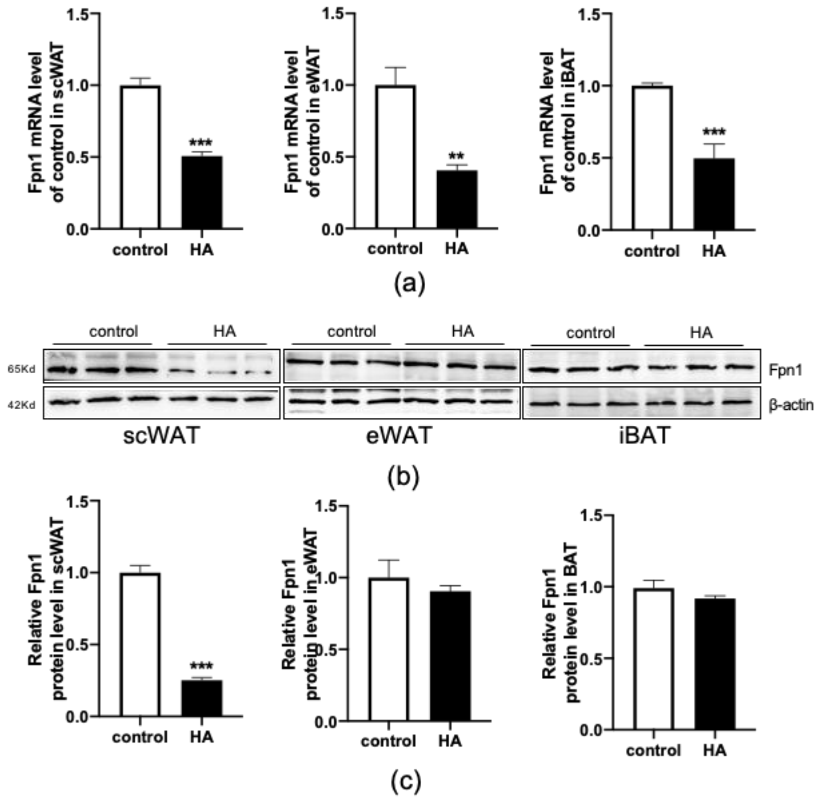

Figure S2. Changes in the mRNA and protein expressions of Fpn1. A RT−qPCR of Fpn1 genes after HA exposure. B Fpn1 protein bands after ECL imaging. C Quantification of the bands, normalized to β-actin. The values are representative of three independent experiments. Data are presented as the mean ± SEM. ** $p < 0.01$, *** $p < 0.001$, versus control.
